# Parallel cyclin E and cyclin A expression in neoplastic lesions of the uterine cervix

**DOI:** 10.1038/sj.bjc.6603038

**Published:** 2006-03-14

**Authors:** F Erlandsson, H-S Martinsson-Ahlzén, K-L Wallin, A-C Hellström, S Andersson, A Zetterberg

**Affiliations:** 1 Cancer Center Karolinska, CCK R8:04, Karolinska University Hospital Solna, Karolinska Institutet, Stockholm 17176, Sweden; 2 Department of Molecular Medicine and Surgery, CMM L8:02, Karolinska Institutet, Center of Molecular Medicine, Karolinska University Hospital Solna, Stockholm 17176, Sweden; 3 Department of Gynecologic Oncology, Radiumhemmet, Karolinska University Hospital, Karolinska Institute, Stockholm 17176, Sweden; 4 Institute for Clinical Science, Intervention and Technics, Division of Obstetrics and Gynaecology, Karolinska University Hospital, Huddinge, Karolinska Institutet, Stockholm 14186, Sweden

**Keywords:** cyclin E, cervical carcinoma, cell cycle

## Abstract

Cyclin E levels are high during late G1 and early S-phase in normal cells. The cyclin E expression over the cell cycle in tumours is not fully known. The impact on patient outcome by high cyclin E levels during other parts of the cell cycle than late G1- and early S-phase is unknown. We set out to study the expression of cyclin E over the cell cycle in cervical carcinomas. Using immunofluorescence staining of cyclin A, digital microscopy, and digital image analysis, we determined which cells in a tissue section that were in S- or G2-phase. M-phase cells were detected by morphology. By simultaneously staining for cyclin E, we investigated the variation in cyclin E levels over the cell cycle in cervical carcinoma lesions. In a case–control study, in which each deceased patient was matched with a patient still alive and well after >5 years of follow-up, we found that the deceased patients had a considerably higher fraction of cyclin A-positive cells staining for cyclin E than the survivors (*n*=36). We conclude that parallel cyclin E and cyclin A expression is an indicator for poor outcome in cervical carcinomas. In addition, we investigated the expression pattern of cyclin E and cyclin A in consecutive biopsy samples from cervical carcinomas at different stages, as well as in human papillomavirus positive or negative adenocarcinomas in order to further study the cyclin E and cyclin A expression pattern in neoplastic lesions of the uterine cervix.

Cervical carcinoma is the third most common malignant disease in women worldwide. Approximately 370 000 new cases are detected annually (Parkin *et al*, 1999). Two main histological types of cervical carcinomas exist, squamous cell carcinoma (SCC) and adenocarcinoma (AC). Squamous cell carcinoma is more common (80%) than AC (15%), and probably develops almost exclusively under the influence of human papillomavirus (HPV) infection (Bohmer *et al*, 2003), whereas 30% of the AC tumours lack HPV involvement (Andersson *et al*, 2003). The incidence of SCC has dropped in the last decades in the developed countries owing to successful screening programmes. Approximately 40% of patients diagnosed with cervical carcinoma in the developed countries die from their disease. The main predictors for outcome include stage, grade, and age at the time of diagnosis.

Cyclin E is a member of the cyclin protein family originally discovered in 1983 (Evans *et al*, 1983). The primary characteristics of the cyclins are their cell-cycle-specific expression patterns, and their association with and subsequent activation of cyclin-dependent kinases (CDKs). Cyclin E levels begin to rise in mid-G1, peak during late G1, and drop off around the G1/S border (Ekholm *et al*, 2001). Cyclin E associates with CDK2. The normal cell progresses through G1 by passing through the G1 restriction point in response to extracellular signals during early G1. Cyclin E then accumulates leading to high cyclin E-dependent CDK2 kinase activity. Cyclin A synthesis then ensues, which leads to S-phase entry. In S-phase, cyclin E is quickly degraded, whereas the cyclin A levels continue to increase throughout S and G2.

A link between overexpression of cyclin E and transformation has been established in model systems (Keyomarsi and Pardee, 1993; Bortner and Rosenberg, 1997). The studies do, however, not address whether it is the increased amount of cyclin E, the presence of aberrant isoforms of cyclin E, or the heterochronic expression (the expression of cyclin E during other phases of the cell cycle than late G1 and early S-phase) of cyclin E that drives transformation. There are also similarly unspecific studies on the clinical implications of cyclin E overexpression (Porter *et al*, 1997; Keyomarsi *et al*, 2002). The presence and clinical implications of high cyclin E levels in tumour cells in late S-phase and/or G2-phase have not been possible to study with the methodology used previously.

Using immunofluorescence staining, digital microscopy, and image analysis techniques, we set out to investigate the clinical implications of the heterochronic cyclin E expression pattern occurring in cervical carcinoma lesions. We have previously shown that cyclin A staining above the minimal detectable level is a dependable marker for cells in S- or G2-phase (Erlandsson *et al*, 2000). By parallel determination of the levels of cyclin E and cyclin A in individual cells from cervical lesions, we were able to detect the presence of abnormally high cyclin E levels in cells containing cyclin A, that is, in cells likely to be in S- or G2-phase.

## MATERIALS AND METHODS

Many of the experimental procedures used, such as the immunofluorescence staining protocol, controls, and image acquisition, have been previously described in detail (Erlandsson *et al*, 2003).

### Selection and acquisition of tumour biopsies

Three different groups of patients were studied. The importance of the cyclin E expression during S-phase for survival was studied in a group of 36 patients treated for cervical carcinoma stage Ib or IIa at the Department of Gynecological Oncology, Radiumhemmet, Karolinska Hospital between 1994 and 1997. Of the patients, 18 died within 13 years after the date of diagnosis, and each of these were matched with a survivor patient with respect to age, stage, grade, lymph node status, and treatment, but which was considered disease free after treatment and more than 5 years of follow-up. We also tried to keep tumour size at diagnosis, smoking habits, parity, and the prior usage of hormonal replacement therapy as similar as possible between the deceased and the survivors. The stage of transformation that cyclin E deregulation occurs at was investigated using multiple biopsy samples from 13 patients treated at the Umeå University Hospital between 1982 and 2000 for cervical lesions at different stages. Two or more biopsies at different stages of the disease were available from each patient. The cyclin E expression pattern in AC of the uterine cervix was studied using tumour samples from 19 selected patients from a larger previously described set of tumours (Andersson *et al*, 2001). The study has been approved by Institutional Review Boards and Ethical Committees from various participating hospitals.

### Handling and staining of tissue sections

Routinely handled cervical biopsies acquired before treatment were routinely fixed in formalin and embedded in paraffin. Sections (4 *μ*m thick) were cut from each tumour, and attached to glass slides. The first and last sections were subjected to histopathological evaluation to confirm tumour status. The slides were stored at −20°C, and rehydrated through a ladder of graded alcohols before staining. Antigenic recovery was performed through microwave cooking for 3 × 5 min in a 0.1 M citrate buffer at pH 6.0. The slides were then washed in washing buffer (0.3 mM NaCl and 0.02% Tween 20 in a buffer consisting of 0.05 mM Tris-HCl at pH 7.6) for 10 min, followed by incubation for 15 min in blocking buffer (1% bovine serum albumin and 0.5% Tween 20 dissolved in phosphate-buffered saline). Thereafter, they were incubated with the primary antibodies diluted in blocking buffer for 48 h at room temperature (RT). Unbound and nonspecifically bound antibodies were removed by washing in washing buffer for 3 × 20 min. To block nonspecific binding of the secondary antibodies, the slides were incubated with 4% donkey serum diluted in blocking buffer for 30 min. The secondary antibodies, diluted in 4% donkey serum in blocking buffer, were added during incubation for 30 min at RT. The coverslips were then washed in washing buffer for 3 × 20 min. Finally, the coverslips were mounted for fluorescence microscopy in H-1200 mounting medium containing 2-(4-amidinophenyl)-6-indolecarbamidine dihydrochloride (DAPI) (Vector Laboratories Inc., Burlingame, CA, USA).

The primary antibodies used were the cyclin E monoclonal antibody HE12 and the cyclin A rabbit polyclonal antibody H-432 (Santa Cruz Biotechnology, Santa Cruz, CA, USA). The secondary antibodies included a fluorescein isothiocyanate (FITC)-conjugated anti-rabbit antibody, and a cyanine 3-dUTP (Cy3)-conjugated anti-mouse antibody (Jackson ImmunoResearch Labs Inc., West Grove, PA, USA). Subsequently, the staining protocol resulted in cell nuclei stained with DAPI, cyclin A stained with FITC, and cyclin E stained with Cy-3. Only tumour samples exhibiting a reliable staining result were used in the further analysis.

### Image acquisition and analysis

Images of approximately 1000–3000 cells from two to five discrete areas from each slide were acquired using a Delta Vision system (Applied Precision Inc., Issaqua, WA, USA) equipped with a monochrome water-cooled CCD camera (Photometrics Ltd, Tucson, AZ, USA). A Plan-Neofluar 63x/NA1.40 lense was used (Carl Zeiss, Switzerland), resulting in images with a resolution of 0.2 *μ*m. The built-in Delta Vision calibration system was applied, which compensated for any nonlinearity in the CCD elements or any consistently uneven light distribution in the microscope. Each image acquisition involved taking three photos, with the optical filter sets for detection of DAPI, FITC, and Cy-3, respectively. Controls were used to exclude any possibility of antibody crossreactions or imperfections in the used optical filters. The tumour areas with the highest percentage of cells staining for cyclin A (i.e., with the highest proliferation) were preferentially imaged.

Image analysis was performed using the IMP image processing software. Cell nuclei were manually segmented in the DAPI image. The segmentation was then applied to the FITC and Cy3 images and the amount of fluorescence emitted from each of the fluorophores from each individual nucleus could then be calculated. M-phase cells were identified based on the nuclear morphology and investigated separately. Only staining arising from cell nuclei was considered.

The intensity of the detected fluorescence signal is directly proportional to the amount of fluorophore present in the specimen (Schauenstein *et al*, 1978; Boddeke, 1999). Controls in which either the monoclonal or polyclonal primary antibody was excluded during the staining proved that there was no detectable crosstalk between the FITC and Cy3 channels (data not shown). We therefore conclude that the measured fluorescence can be assumed to be linear to the levels of the stained antigen. As both cyclin E and cyclin A staining is fairly homogeneously distributed throughout the nucleus, the average fluorescence detected per pixel can be assumed to represent a semiquantitative measurement of the nuclear concentration of cyclin E and cyclin A. The measured cyclin E and cyclin A levels can then be compared between neighbouring cells, but not between cells from different tumours.

In order to determine which cells stained positive for cyclin E and/or cyclin A in each studied tumour area, we needed to set a threshold level for each stain above which the measured fluorescence would mark the cell as positive. An algorithm previously described (Erlandsson *et al*, 2003) based on density kernel estimates was used to automatically set objective thresholds that separated the negative population of cell nuclei from those actually containing cyclin E and/or cyclin A. In brief terms, the algorithm creates a function that approximates the distribution of the measurements as plotted in a histogram. Maxima in the second derivative of the function are then calculated and proposed as thresholds. However, in highly proliferative tumours, the negative population was occasionally too small to be reliably detected by the algorithm, and the thresholds were then manually set by visual inspection of the original images, although aided by secondary threshold suggestions by the algorithm.

## RESULTS

### A high fraction of S or G2 cells containing cyclin E is associated with poor prognosis

The results of the clinical data of 36 patients (survivor/deceased) with SCC are shown in Table 1. Half of the patients died within 3 years from the date of diagnosis, whereas the other half survived and was considered disease free after more than 5 years of follow-up without recurrence. The selected tumours were stained for cyclin A and cyclin E and photographed. Using image analysis methods described above, the nuclear content of the individual cells was calculated. Cells staining above the threshold level for cyclin A were considered to be cells in S- or G2-phase. The fraction of these cells staining for cyclin E above the threshold level was calculated for each tumour. The average is shown for each patient in Table 1. In 15 out of 18 patient pairs, the deceased patient had a higher fraction of cyclin A-positive cells staining for cyclin E than the surviving patient did, which makes the difference in expression patterns statistically significant (*P*<0.002). The results indicate that a parallel expression of cyclin E and cyclin A is correlated to poor outcome. Carcinomas of the uterine cervix containing cells that simultaneously express cyclin E and cyclin A are more likely to kill the host than the tumours that do not.

**Figure 1:**
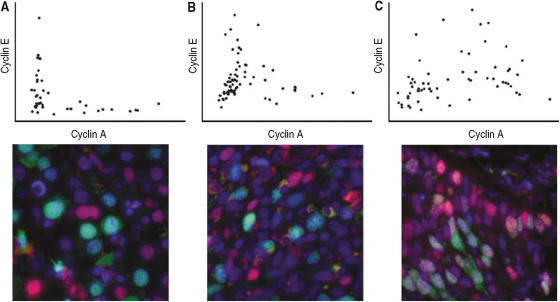
At least three different patterns of cyclin E and cyclin A expression over the cell cycle could be distinguished. All three plates (**A**–**C**) come from cervical carcinoma lesions, although plate A is very similar to the pattern seen in normal or inflamed cervical tissue. Each dot in the plots represents the cyclin A and cyclin E measurements from one cell nucleus in the image shown underneath. Cells with higher cyclin A content are cells in S- or G2-phase. Cells with low cyclin A and cyclin E contents are cells in early G1, whereas the cells with a low cyclin A content and a high cyclin E content are either senescent cells or cells in late G1. In the images, DAPI-stained nuclei are blue, cyclin A is green, and cyclin E is red. In plate A, all cells containing cyclin E are senescent or in late G1. In plate B, some cells in early S-phase retain an intermediate amount of cyclin E before degrading it in G2 (G2 cells have the highest cyclin A content). In the tumour shown in plate C, cyclin E is primarily present in S- and G2-cells, and is expressed throughout S-phase.

Figure 1 shows the cyclin A staining intensity plotted *vs* the cyclin E staining intensity in three different tumours. It is evident from the figure that these three tumours display highly different cyclin E expression patterns. Calculating the fraction of cyclin A-positive cells that stain positive for cyclin E does not always differentiate between the types of patterns shown in Figure 1B and C. We therefore assumed that a fraction of cells staining strongly for cyclin E throughout S-phase is a more dangerous phenotype than having a fraction of cells staining for cyclin E only in early S-phase, and then set out to visually compare the staining patterns within each matched patient pair. Two independent investigators (FE and AZ) could in a double-blind set-up correctly tell which patient died in 15/16 out of the 18 pairs (*P*<0.002, *P*<0.0001) simply by visually evaluating plots as in Figure 1. The pairs in which the tumours exhibited similar percentages of cyclin E staining cells among the cyclin A-positive cells were the pairs that were the hardest to separate also by visual evaluation, indicating that the two methods of evaluation yielded similar results.

### Predictive value of only cyclin E or only cyclin A staining

Table 1 lists the fraction of cells staining for cyclin E out of all cells in each tumour. The percentages ranged from 12 to 34%, and in only 10 out of the 18 pairs did the deceased patient have a higher fraction of cells staining for cyclin E than the survivor. The percentage of cells staining for cyclin A out of all cells ranged from 3 to 39%, and in nine out of the 18 pairs did the deceased patient exhibit a higher fraction (i.e., higher proliferation) than the survivor. Therefore, we conclude that in our small study, we could not find any significant difference in the overall expression of cyclin E between deceased and survivors. Neither could we find a significant difference in proliferation, measured as fraction of cells staining for cyclin A, between the deceased and the survivors. However, the averages for cyclin E and cyclin A staining were slightly higher in the deceased patients (Table 1, bottom right), which indicates a tendency that would probably become significant in a larger study.

### Cyclin E could not be detected in M-phase cells

**Figure 2:**
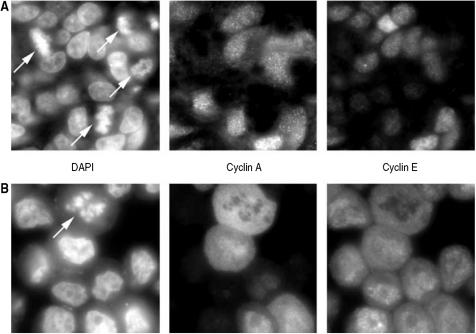
Cyclin E was degraded before mitosis in all investigated cervical carcinoma lesions. Plate A shows an example of a tumour with four M-phase cells (marked by arrows). Neither of them stain positive for cyclin E. Plate B shows cells transfected with CCNE, the cyclin E gene, which express high levels of cyclin E throughout the cell cycle. Also, M-phase cells transfected with cyclin E exhibit a clear cyclin E staining, which excludes the possibility of a methodological error.

As the presence of cyclin A-positive cells with high cyclin E levels was found to be clinically relevant, we also wanted to investigate the cyclin E levels during mitosis. By visual identification of cells containing condensed chromosomes, the cells in M-phase could be investigated in more than 50 squamous carcinomas, as well as in 17 ACs, from the uterine cervix. Not one single M-phase nucleus containing cyclin E could be found throughout all of the studied tumours. An example of a tumour with a highly aberrant cyclin E expression pattern is shown in Figure 2A. Virtually all cells staining for cyclin A also stains for cyclin E, but the M-phase cells do not. This indicates that although the tumour cells commonly fail to degrade cyclin E during S and/or G2, they succeed to do so before M-phase entry.

### The development of cyclin E aberrations in consecutive progressing lesions

We also attempted to study when in the transformation process that cyclin E begins to appear in parallel with cyclin A. Tissue samples containing everything between normal or inflamed epithelium to invasive squamous carcinoma, including cervical intraepithelial neoplasia (CIN) and carcinoma *in situ* lesions, from 11 different patients were studied. None of the studied biopsies containing normal or inflamed epithelium exhibited any areas where cyclin A-positive cells contained cyclin E at significant levels. One of the invasive carcinomas exhibited gravely aberrant expression pattern. However, the highest expression of cyclin E among cyclin A-positive cells in an invasive lesion was 24%, similar to the expression in the CIN2 lesion biopsied 55 months prior exhibited (25%).

### Cyclin E expression pattern in ACs of the uterine cervix

In order to investigate the frequency of heterochronic cyclin E expression in ACs of the uterine cervix, we stained and evaluated tumour tissue from 17 patients. Similar to the studied SCCs, the ACs exhibited highly varying levels of cyclin E in cells expressing cyclin A. Also in ACs not infected by HPV did we find highly aberrant cyclin E expression patterns, indicating that HPV infection is not a prerequisite for parallel cyclin E and cyclin A expression. In the tumours from deceased patients, the percentages were 15, 25, 31, and 37%, rendering an average of 27%, which was not significantly different from the 24% average among the survivors (*P*=0.52).

## DISCUSSION

In recent years, the expression pattern of cyclin E has acquired increased interest as deregulated expression of cyclin E potentially could result in genomic instability, and thereby tumour progression. We used carefully selected tumour biopsies, immunofluorescence staining, digital image acquisition, and image analysis in order to study the clinical implications of a parallel expression of cyclin E and cyclin A, most likely caused by a heterochronic cyclin E expression (the expression of cyclin E during S and G2). We found that cyclin E was commonly expressed in parallel with cyclin A, both in squamous carcinomas and in ACs of the uterine cervix, and that the degree of the aberration was related to patient survival. We could not detect a single tumour cell in M-phase containing cyclin E, which indicates that cyclin E is consistently degraded before M-phase entry in tumours of the uterine cervix.

The study of the cyclin E expression pattern *in vivo* puts high demands on the methodological approach. We choose to rely on the expression of cyclin A to determinate the position in the cell cycle of each investigated cell, as we have previously shown that cyclin A reaches detectable levels at the G1/S border, and continues to accumulate until M-phase both in normal and transformed cells (Erlandsson *et al*, 2000). M-phase cells were identified solely based on morphology. The main disadvantage to using cyclin A staining as a marker of cell cycle position is that image analysis techniques must be utilised in order to reach an acceptable level of reliability, which makes the evaluation process tedious and time consuming. Therefore, we had to design our study for maximum efficiency. Progress in the near future may automate parts of the process, such as image acquisition, preprocessing, segmentation, and data extraction. Consistent tissue-handling routines, and more robust antibodies would also speed up and simplify the evaluation of the cyclin E expression over the cell cycle.

Our main result is that parallel expression of cyclin E and cyclin A is clinically significant, and may be a marker for poor prognosis. Others have previously seen an association between cyclin E overexpression and survival, either using Western blotting (Porter *et al*, 1997; Datta *et al*, 2000; Keyomarsi *et al*, 2002), histochemistry (Porter *et al*, 1997; Datta *et al*, 2000; Keyomarsi *et al*, 2002), or flow cytometry (Darzynkiewicz *et al*, 1996; Nielsen *et al*, 1999). Our study is fundamentally different from those previous studies, as we neither studied the total amount of cyclin E in the tissue nor just the number of cells staining for cyclin E. Instead, we focused on how cyclin E is expressed in relation to cyclin A expression, which is likely to be related to how cyclin E is expressed over the cell cycle. Our results fall well in line with those previous studies, as cyclin E expression during a larger part of the cell cycle often will lead to an increased total amount of cyclin E in the tissue, as well as an increased fraction of cells staining for cyclin E. However, in the search for the link between cyclin E expression and clinical outcome, it is crucial to know whether it is the heterochronic expression of cyclin E, rather than the amount of cyclin E in the tissue, that causes the genetic instability.

There are several potential explanations to why a high cyclin E level in parallel with cyclin A expression may result in a more dangerous tumour. It has previously been suggested that heterochronic expression of cyclin E can cause chromosomal aberrations in cells transfected with the cyclin E1 gene CCNE (Spruck *et al*, 1999). Furthermore, the accumulation of cyclin E in Skp2−/− mice was associated with an increase in centrosome aberrations and polyploidy (Nakayama *et al*, 2000). Therefore, heterochronic expression of cyclin E has been proposed to cause aneuploidy, which is known to be associated with poor outcome in human cancers. However, in squamous carcinoma of the uterine cervix, virtually all tumours are aneuploid. Our findings that a parallel expression of cyclin E and cyclin A is related to poor outcome is therefore likely to be caused by some other mechanism or process than just the precipitation of aneuploidy by cyclin E expression throughout the cell cycle.

Recent *in vitro* studies have shown that high cyclin E levels throughout the cell cycle may result in the construction of defective pre-replication complexes in early G1 (Ekholm-Reed *et al*, 2004a). This mechanism does, however, seem unlikely to play a big part in cervical carcinomas, as we show that M-phase cells, and subsequently also cells in early G1, do not contain any cyclin E. In more general terms, our findings that the levels of cyclin E rarely or never are completely free of cell cycle control *in vivo* in cervical carcinomas raise some questions regarding the applicability of transfection studies *in vitro* in which cell cycle-regulated proteins are ubiquitously expressed in order to mimic the situation in tumours. However, the generality of our M-phase data is questioned by a study proving that cyclin E can, at least in rare cases, appear in M-phase cells in human endometrial tumours (Ekholm-Reed *et al*, 2004b).

There are two more possible explanations to our findings, which we have failed to control for. If cyclin A begins to accumulate prematurely, for instance, owing to oncogenic activation in the signalling cascades, then the cyclin E peak which usually occurs in late G1 might instead occur during an S-phase triggered prematurely by cyclin A. Premature S-phase entry could then cause a defective DNA replication, and the parallel cyclin E and cyclin A levels would merely be a symptom, not the cause, of the cell cycle aberration. Finally, our data even paradoxically suggest that the lack of high cyclin E levels during G1 (see Figure 1, plates A, B, and C) could be the cause of the more malignant phenotype of the cervical carcinomas, as the expression pattern in Figure 1, plate C generally was associated with worse outcome than the patterns shown in Figure 1, plates A and B.

The data we present herein indicates that the expression pattern of cyclin E compared to the cyclin A expression pattern over the cell cycle is an important factor for outcome of patients with cervical carcinomas. Today, proliferation markers such as cyclin A, Ki-67, or proliferating cell nuclear antigen are commonly used in routine investigations, but in this paper we show that the cyclin E expression pattern may be a better predictor of outcome. Larger cohort studies will naturally have to be performed before the introduction of the method in routine clinical praxis. Furthermore, future mechanistic studies revealing the true nature of the connection between cyclin E overexpression and patient survival are highly desirable, as they have the potential to uncover novel therapeutic targets as well as sharpen the currently used rather blunt diagnostic and prognostic tools.

## Figures and Tables

**Table 1:** The matching of the included patients, and the resulting cyclin E and cyclin A staining fractions

								**Cyc E+ of cyc A+**				
**Outcome**	**Smoker**	**HRT**	**Parity**	**Age**	**Stage**	**Size**	**Grade**	**Aver (%)**	**Low (%)**	**High (%)**	**E+ (%)**	**A+ (%)**
Survivor	Y	N	2	49	Ib	1	2	16	13	17	16	25
Deceased	N	N	3	50	Ib	2	2	20	18	20	17	18
Survivor	Y	N	3	49	Ib	4	1	17	15	20	16	22
Deceased	Y	N	4	48	Ib	5	2	26	26	27	16	39
Survivor	Y	N	3	35	Ib	1	2	15	11	23	5	3
Deceased	N	N	3	37	Ib	2	3	27	24	31	16	22
Survivor	N	N	0	42	Ib	3	2	19	18	20	16	25
Deceased	N	N	1	42	Ib	4	2	26	24	28	11	21
Survivor	Y	N	1	31	Ib	2	1	12	5	16	18	30
Deceased	N	N	2	32	Ib	4	1	18	17	18	21	29
Survivor	?	N	1	48	Ib	2	1	16	14	17	8	14
Deceased	Y	Y	3	49	Ib	4	2	25	24	26	15	32
Survivor	Y	N	3	41	Ib	2	1	26	25	27	11	30
Deceased	N	N	4	36	Ib	0	1	12	6	23	22	25
Survivor	Y	N	0	33	Ib	5	1	29	24	36	7	10
Deceased	N	N	2	30	IIa	3	1	15	7	23	23	19
Survivor	N	N	2	35	IIa	7	1	18	16	20	19	26
Deceased	Y	N	2	36	IIa	4	1	23	23	23	18	13
Survivor	Y	N	3	54	IIa	2	1	30	29	31	17	13
Deceased	?	N	3	57	IIa	2	2	14	11	16	20	23
Survivor	N	N	1	40	Ib	4	1	20	10	32	13	18
Deceased	?	N	5	39	Ib	2	2	27	26	28	9	29
Survivor	N	N	3	42	Ib	1	2	23	16	29	16	30
Deceased	N	N	2	38	Ib	1	1	34	29	37	13	36
Survivor	Y	N	3	30	IIa	4	2	19	12	25	13	35
Deceased	Y	N	1	38	IIa	4	1	29	26	31	15	29
Survivor	?	N	4	57	Ib	2	2	4	2	6	31	36
Deceased	Y	N	2	60	Ib	5	1	31	25	37	14	26
Survivor	N	Y	3	55	Ib	2	2	25	12	32	14	25
Deceased	N	Y	0	51	Ib	4	1	26	20	33	15	20
Survivor	N	N	2	52	Ib	3	1	17	3	24	27	21
Deceased	N	N	2	54	Ib	4	1	26	21	29	21	23
Survivor	Y	Y	2	49	Ib	2	2	13	7	20	13	27
Deceased	Y	N	0	44	Ib	5	1	20	14	23	16	30
Survivor	N	N	3	36	Ib	2	1	16	11	19	18	32
Deceased	N	N	2	39	Ib	3	1	25	25	27	16	21
Survivors			2.2	43.2		2.9		18.6			15.4	23.4
Deceased			2.3	43.3		3.2		23.6			16.5	25.3

HRT=hormone replacement therapy.

The column ‘Cyc E+ of cyc A+’ exhibits the fraction of cells staining for cyclin A (i.e., cells in S- or G2-phase) that were found to stain for cyclin E. ‘Aver’ contains the average throughout the tumour. During image acquisition, two to five different areas containing roughly 1000 cells each were photographed from each tumour. When each area was analysed individually, there were some variations between the areas within the same tumour, hence ‘low’ and ‘high’ shows the fractions in the areas with the least and the highest cyclin E staining out of the studied areas in the tumour. The data show that intratumoural variations in the expression pattern of cyclin E exist, indicating that tumours develop different clones with varying degrees or varying mechanisms of disturbed cell cycle control. ‘Size’ contains the tumour size measured in centimetres, and ‘grade’ shows the tumour differentiation grade, 1=poorly differentiated, 3=moderately, 5=well differentiated. The columns A+ and E+ exhibit the fraction of all cells staining positive for cyclin A and cyclin E, respectively.
